# Antitumor effect of poly lactic acid nanoparticles loaded with cisplatin and chloroquine on the oral squamous cell carcinoma

**DOI:** 10.18632/aging.202297

**Published:** 2020-12-11

**Authors:** Qiang Li, Xia Liu, Wei Yan, Yong Chen

**Affiliations:** 1 Department of Oral and Maxillofacial Surgery, Cangzhou Central Hospital, Cangzhou, Hebei Province, China; 2 Department of Basic Nursing, Cangzhou Medical College, Cangzhou, Hebei Province, China

**Keywords:** poly lactic acid, transmission electron microscopy, dynamic light scattering, oral squamous cell carcinoma

## Abstract

Purpose: Poly lactic acid (PLA) combined with cisplatin-chloroquine nanoparticles (CDDP/CQ-PLA NPs) and PLA combined with cisplatin nanoparticles (CDDP-PLA NPs) were prepared to investigate their inhibitory effects on the proliferation of oral squamous cell carcinoma (OSCC) Cal-27cell line.

Patients and methods: We prepared CDDP/CQ-PLA NPs and CDDP-PLA NPs. Transmission electron microscopy (TEM) and dynamic light scattering (DLS) were used to detect the physiological characteristics and particle size parameters of drug-loaded nanoparticles. The drug concentration and cumulative release were measured by UV and visible spectrophotometer. MTT assay was used to detect viability of Cal-27 cells. Annexin/PI staining was used to detect cell apoptosis. Biological kits were used to detect malondialdehyde (MDA) content, catalase (CAT) activity, antioxidant enzyme superoxide dismutase (SOD) activity and glutathione peroxidase (GSH PX) activity in Cal-27 cells. Western blot was used to detect apoptosis and autophagy of Cal-27 cells.

Results: CDDP/CQ-PLA NPs and CDDP -PLA NPs had good drug loaded nanoparticles and drug release. CDDP/CQ-PLA NPs showed higher ROS and apoptosis rate, and lower autophagy than CDDP-PLA NPs.

Conclusion: CDDP/CQ-PLA NPs reduced autophagy and enhanced ROS and apoptosis of Cal-27 cells, which shows a potential in the clinical treatment of OSCC.

## INTRODUCTION

Oral squamous cell carcinoma (OSCC) is a common malignant tumor in the oral cavity [1]. At present, it is mainly treated by surgical resection, followed by postoperative chemotherapy and radiotherapy [2]. Although the treatment of oral cancer has been gradually improved, the 5-year survival rate of patients with oral cancer has not been significantly improved [3]. Cisplatin, as a first-line chemotherapy drug for oral cancer, has many disadvantages, such as drug resistance, irregular absorption *in vivo*, short half-life and obvious side effects [4, 5]. Therefore, it is urgent to improve the sensitivity of oral squamous cell carcinoma to cisplatin, prolong the effective concentration of cisplatin *in vivo* and reduce the side effects.

Nano drug has become a promising alternative to traditional small molecule chemotherapy [6, 7]. As a kind of nanoparticles, PLA nanoparticles can target tumor passively or actively without being cleared by the body [8]. The design of lactate nanoparticles loaded with chemotherapy drugs can effectively target tumor therapy and overcome multidrug resistance [9]. This new strategy based on PLA nanoparticles can enhance the therapeutic effect of tumor chemotherapy and reduce the side effects of the body compared with the traditional strategy of non-targeted drug combination [10].

Autophagy is a kind of self-phagocytosis phenomenon that widely exists in eukaryotic cells [11]. It is the response of cells to endogenous or exogenous stimulation, and it participates in the process of degradation and recycling of organelles and intracellular macromolecular proteins [12]. At present, autophagy is closely related to the occurrence and development of tumors, and it has become a hot topic in the field of tumor research [13]. It is found that inhibition of autophagy can effectively inhibit the growth of OSCC [14]. In this study, we found that polylactide nanoparticles loaded with cisplatin and chloroquine (CDDP/CQ-PLA NPs), an autophagy inhibitor, have excellent *in vitro* release characteristics, and inhibit the proliferation of OSCC by promoting oxidative stress and apoptosis of cancer cells.

## RESULTS

### Synthesis and characterization of CDDP/CQ-PLA NPs

**Figure 1:**
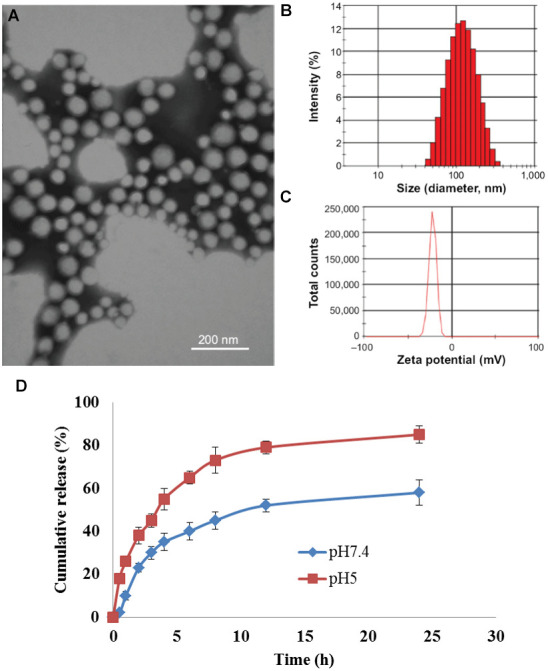
**Synthesis and characterization of CDDP/CQ-PLA NPs.** (**A**) TEM image; (**B**) Dynamic light scattering measurements; (**C**) Zeta potential; (**D**) drug release kinetics at 0.5 h, 1 h, 2.5 h, 4 h, 4.5 h, 6 h, 8.5 h, 11.5 h and 24.5 h.

To examine the functional nanoparticles, we performed a series of characterization assays. From the TEM image, we found that the diameter of CDDP/CQ-PLA NP was about 20 nm (Figure 1A). Zeta potential and dynamic light scattering measurements were further conducted and confirmed successful synthesis of CDDP/CQ-PLA NP (Figure 1B, 1C). The drug release kinetics showed that CDDP/CQ-PLA NPs were released faster in the acid condition (pH = 5) than neutral condition (pH = 7) (Figure 1D).

### CDDP/CQ-PLA NPs reduces the viability of OSCC cells

**Figure 2:**
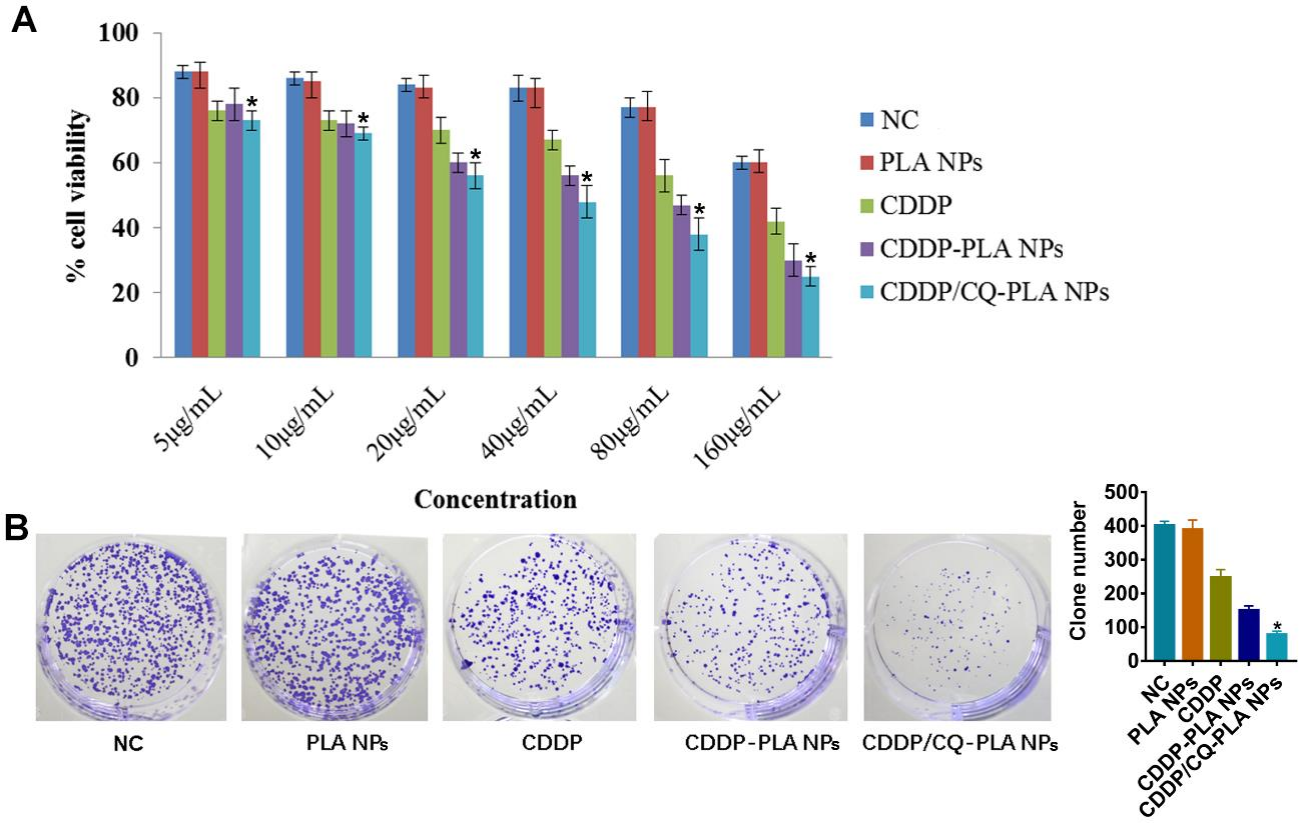
**CDDP/CQ-PLA NPs reduces the viability.** (**A**) Cell viabilities in CAL-27 using MTT assay. (**B**) Colony forming assays with a concentration of 40μg/mL. *P < 0.05. NC: PBS.

To evaluated the cytotoxicity on OSCC cell. We treated CAL-27 cells with different concentrations of CDDP/CQ-PLA NPs (0, 5, 10, 20, 40, 80, 160 ug/ml). The result of MTT and colony formation showed that CDDP/CQ-PLA NPs had more cytotoxicity than CDDP -PLA NPs and CDDP. (Figure 2A, 2B and Supplementary Figure 1).

### CDDP/CQ-PLA NPs induces caspase-dependent apoptosis in OSCC cells

**Figure 3:**
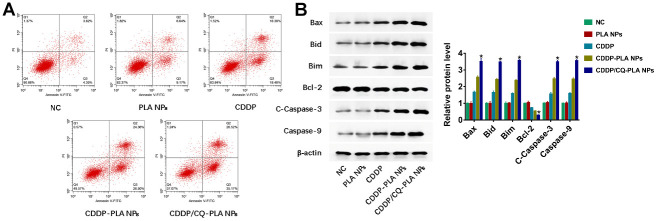
**CDDP/CQ-PLA NPs induces caspase-dependent apoptosis.** (**A**) Cell apoptosis in CAL-27 using Annexin-V/PI assay; (**B**) Western blot of Bax, Bid, Bim, Bcl-2, Caspase-3, and Caspase-9, β-actin as reference. *P < 0.05, NC: PBS.

In order to explore the action of CDDP/CQ-PLA NPs’ cytotoxicity. We examined apoptosis and ROS. As shown in Figure 3, CDDP/CQ-PLA NPs leaded to more apoptosis than CDDP -PLA NPs and CDDP through active caspase-3 (Figure 3A), which was confirmed by western blot (Figure 3B and Supplementary Figure 2) and indicated that CDDP/CQ-PLA NPs induces caspase-dependent apoptosis in OSCC cells.

### CDDP/CQ-PLA NPs induces oxidative damage in OSCC cells

**Figure 4:**
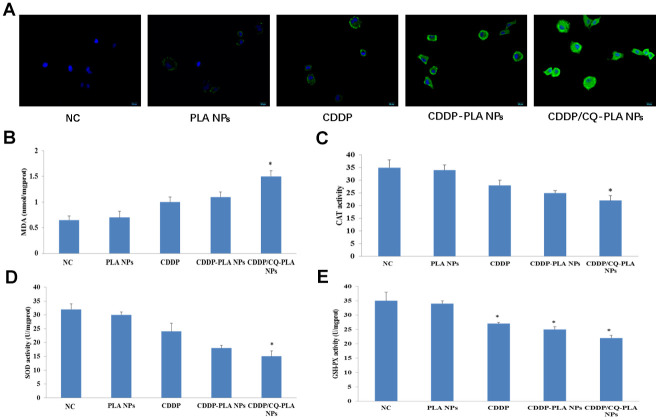
**CDDP/CQ-PLA NPs induces oxidative damage.** (**A**) ROS production; (**B**) MDA activity; (**C**) CAT activity; (**D**) SOD activity; (**E**) GSH-P activity; Data are mean ± SD; *P < 0.05, **P< 0.01, NC: PBS.

Furthermore, we found that CDDP/CQ-PLA NPs induced more ROS production (Figure 4A and Supplementary Figure 3) and MDA (Figure 4B), followed by decreased CAT (Figure 4C), SOD (Figure 4D), and GSH-P activity (Figure 4E), which suggested that CDDP/CQ-PLA NPs induces caspase-dependent apoptosis through oxidative damage in OSCC cells.

### CDDP/CQ-PLA NPs reduces autophagy in OSCC cells

**Figure 5:**
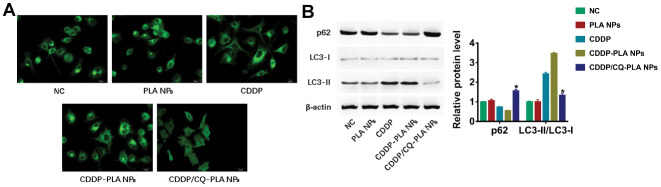
**CDDP/CQ-PLA NPs reduces autophagy.** (**A**) Immunofluorescence confocal image of LC3; (**B**) Western blot of p62, LC3-I, and LC3-II, actin as reference. *P < 0.05, NC: PBS.

As CQ is an autophagy inhibitor, we examined the autophagy change caused by CDDP/CQ-PLA NPs. Confocal image show that CDDP/CQ-PLA NPs reduced the GFP-LC3 puncta caused by CDDP or CDDP -PLA NPs (Figure 5A and Supplementary Figure 4A). Western blot showed the consistent results that CDDP/CQ-PLA NPs reduced LC3-II and induced p62 protein expression (Figure 5B and Supplementary Figure 4B), which indicated that CDDP/CQ-PLA NPs inhibits autophagy in OSCC cells.

## DISCUSSION

Every year around the world, about 300000 people suffer from OSCC [15, 16]. With the advent of new chemotherapeutics in recent years, and the improvement of drug delivery methods and routes, chemotherapy has become one of the important means for the treatment of OSCC, which is often used to reduce the volume of lesions for surgical resection [17, 18]. Cisplatin is a first-line chemotherapeutic drug for clinical use. The platinum atom is cross linked with DNA strand, showing cytotoxic effect. As a new drug carrier, lactate nanoparticles have been used to support and control the release of anticancer drugs in recent years.

In recent years, the development of nanotechnology provides a new technology path for anti-tumor treatment. PLA nanoparticles can be completely degraded and absorbed in the body, without residue and side effects [19]. The lactate formed by degradation in the body participates in the tricarboxylic acid cycle of the human body, and finally becomes CO2 and water. In addition, PLA nanoparticles can target tumor specifically, so it is very suitable for loading chemotherapy drugs for targeted treatment [20, 21]. Autophagy as a survival mechanism can protect tumor cells from the damage of anti-tumor drugs. When tumor cells are exposed to cisplatin, they will produce damaged organelles, damaged proteins and other harmful components. Lots of studies have proved that autophagy can clear the damaged organelles and make tumor cells escape death and survive when cisplatin chemotherapy kills tumor cells [22, 23]. In addition, studies have shown that down-regulation of autophagy can enhance the sensitivity of cisplatin chemotherapy for oral cancer [24]. In accordance with the previous research results, we detected the expression of autophagy related genes LC3 and p62, and found that the autophagy of CDDP-PLA NPs increased, but CDDP/CQ-PLA NPs significantly inhibited the proliferation of tumor.

Reactive oxygen species (ROS) is a general term for the oxygen-containing compounds produced by cells in the process of metabolism, which can regulate the activity of various molecules and signal transduction pathways in cells [25]. Cui Q and other research results show that a small amount of ROS can be used as signal molecules to mediate signal transduction pathways, participate in inflammatory and immune responses, and play a protective role on cells [25]. Under normal physiological conditions, ROS in cells is regulated and maintained in a balanced state by the intracellular antioxidant system. After CDDP enters cells, it can promote the oxidative stress response of cells and lead to the accumulation of ROS in cells by damaging the electron transport chain of mitochondrial respiration. In the body, antioxidant system (SOD, GSH, etc.) can remove excess ROS to maintain the balance of redox reaction. Present results showed that the content of ROS and SOD decreased significantly, while the content of GSH and MDA increased significantly. It is suggested that the combination of drugs can inhibit the activity of antioxidant enzymes, lead to the disorder of intracellular redox level, promote the enhancement of lipid peroxidation, and cause apoptosis.

**Figure 6:**
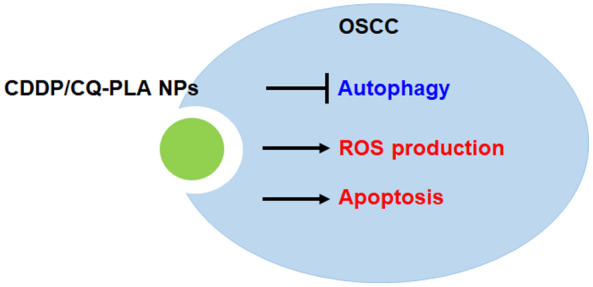
**Proposed signaling mechanism for CDDP/CQ-PLA NPs in OSCC.**

In conclusion, we found that PLA nanoparticles loaded with cisplatin and chloroquine, an autophagy inhibitor, had excellent *in vitro* release characteristics, and inhibited the proliferation of OSCC by promoting oxidative stress and apoptosis of cancer cells. However, the specific mechanism of the drug's inhibition of cancer cell survival through which signal transduction pathway is not clear (Figure 6).

In the future, we plan to further examine the specific mechanisms of autophagy and oxidative stress-related signaling pathways of the drug *in vivo* and *in vitro*. Cancer stem cells (CSCs) are a subgroup of cells with self-renewal and differentiation potential in tumor tissues. The existence of CSCs is the fundamental cause of the failure of traditional treatment and tumor recurrence. Considering the important role of CSC [26], we aimed to explore the effect of CDDP/CQ-PLA NPs on CSC.

## CONCLUSION

In conclusion, CDDP/CQ-PLA NPs has good drug-loaded and drug-release characteristics. In Cal-27 cells, CDDP/CQ-PLA NPs can reduce autophagy and enhance ROS and apoptosis, which shows a potential in the clinical treatment of OSCC.

## MATERIALS AND METHODS

### Materials

Antibody against Bax, Bid, Bim, Bcl-2, caspase-3, caspase-9, p62, LC3, β-actin were purchase from Abcam company.

### Cell culture

OSCC cell line, CAL-27 purchased from ATCC were cultured in DMEM with 10%FBS and 1% penicillin-streptomycin at 37° C, 5% CO2 condition.

### Preparation of CDDP/CQ-PLA NPs

Cisplatin (Sigma, USA) is dissolved in 1 mL of anhydrous methanol, and then the above cisplatin solution and 3mL of PEG-PLA (Sigma, USA) solution dissolved in chloroform are uniformly mixed (the ratio of lipid material to cisplatin is 5: 1)**.** Then the mixed solution of cisplatin and PEG-PLA were transferred into an eggplant bottle, and the organic solvent was removed by a vacuum rotary evaporator to form a dry drug lipid film at the bottom of the bottle. Subsequently, 2mL PBS buffer solution was added to dissolve the drug lipid membrane, and the drug lipid membrane was hydrated in a water bath at 60° C for 30 minutes. The hydrated solution was filtered through polycarbonate membrane (FormuMax Scientific, USA) with a pore diameter of 0.2 micron to obtain cisplatin polylactic acid nanoparticles (CDDP-PLA NPs) solution [27]. CDDP/CQ-PLA NPs were prepared using the same method above using cisplatin and chloroquine instead of cisplatin. PLA NPs were prepared using the same method above using no drug instead of cisplatin.

### Surface morphology, ζ-potential and particle size

Transmission electron microscopy (TEM) and dynamic light scattering (DLS) were used to detect the morphological characteristics and particle size parameters of drug-loaded nanoparticles. The nanoparticle solution was diluted with deionized water. Then we dropped 10 μL of sample onto a carbon-coated copper mesh, dried overnight at room temperature, and volatilized excess water. The sample then was re-dyed with 5 μL of 1% uranyl acetate solution for 30 seconds, and filter paper was used to suck the dyeing solution, the sample was dried at 42° C with a constant temperature dryer. The morphology of the sample was observed with a transmission electron microscope (Tecnai G2 20 STWIN). Different nano micelle solutions were diluted to 0.25 mg/mL with deionized water, and 1mL sample was taken to measure the hydration diameter and Zeta potential of nano particles with a laser particle sizer (Zetasizer 5000) [28]. Determination conditions: laser wavelength 633nm, angle 90, temperature 25° C.

### Drug loading and encapsulation efficiency

DMF was used to dissolve the nanoparticles. The absorbance at 703nm was measured by ultraviolet spectrophotometer for cisplatin content and 330nm for chloroquine content. The following equation was applied:

EE  %=Wt/Ws×100 %

LC  %=Wt/Wo×100 %

Note: EE: encapsulation efficiency; LC: loading content; Wt: Amount of drug encapsulated in nanoparticles; Wo: Initial Dosage of Drugs; Ws: Number of Freeze-dried Nanoparticles.

### Drug release kinetics

4 mg of cisplatin-chloroquine polylactic acid nanoparticles (CDDP/CQ-PLA NPs) were dissolved in the solution, and then put into PBS (pH=5.0) that added HCl and PBS (pH=7.4) to simulate tumor metaacid intracellular body (pH=5.0) and neutral blood environment (pH=7.4), respectively. Then the suspension was transferred into a dialysis bag with a rejection of 3 kDa, and immersed in 20 ml PBS, and incubated at 37° C for 100 rpm under shaking [29]. 5 ml of the release solution was taken out every 1 hour. The absorbance at 703nm was measured with an ultraviolet spectrophotometer for the content of cisplatin, and the absorbance at 330nm was measured with a visible spectrophotometer for the content of chloroquine. At the same time, the same volume of new PBS solution was added to the release medium [30]. The drug concentration and cumulative release were calculated.

### Colony-forming assays/ cytotoxicity study

OSCC cell line CAL-27 was cultured overnight in 96-well plates and treated with the following groups: (1) NC group (PBS); (2) PLA NPs; (3) cisplatin; (4) CDDP-PLA NPs; (5) CDDP/CQ-PLA NPs. Set the concentration range according to cisplatin concentration: 5,10,20,40,80,160 μg/mL. Action time: 24h. At a specific concentration of cisplatin, MTT and colony forming assays were used to verify the different cell viability and proliferation among the groups. At 72 h after treatment, we measured the OD value of MTT (DUOXI, China) and took pictures of colony forming assay.

### Apoptosis analysis

CAL-27 cells were seeded in 6-well plates and attached overnight, then treated with the following groups: (1) NC group (PBS); (2) PLA NPs; (3) cisplatin; (4) CDDP-PLA NPs; (5) CDDP/CQ-PLA NPs. After treatment with 40ug/ml cisplatin and continuous culture for 24h, Annexin/PI staining (Abcam, UK) was used to detect cell apoptosis.

### Assessment of oxidative damage

Biochemical kits were used to detect malondialdehyde (MDA) content, catalase (CAT) activity, antioxidant enzyme superoxide dismutase (SOD) activity and glutathione peroxidase (GSH-Px) activity in CAL-27 cells. Kits can be purchased from Nanjingjiancheng company.

DCFH-DA purchased from Biyuntian company. is used as fluorescent probe and laser confocal microscope is used to detect ROS generation.

### Western blot analysis

Total protein was collected from cells with RIPA lysis Mix (Beyotime, China). The protein extract was separated on SDS-PAGE gel and transferred to nitrocellulose membrane (Pierce, USA). The membrane was sealed with skim milk (Abbexa, UK) and incubated overnight with primary antibody. The membrane was then incubated with horseradish peroxidase-bound IgG (Proteintech, USA). Enhanced chemiluminescence (Pierce, USA) is used to detect signals.

### Immunofluorescence confocal laser microscopy

CAL-27 cells were cultured on coverslips at a density of 5×10^4^ cells/well in 500 μL of complete medium (Hyclone, USA). After treatments, immunofluorescence analyses were carried out as previously described using LC3 antibodies. Cells were examined under an Olympus FV1000 confocal laser microscope (Tokyo, Japan).

### Statistical analysis

Results are expressed as mean values ± standard deviation. Statistical analyses were assessed with Student’s test and one-way ANOVA using GraphPad Prism 7.0 and SPSS19.0. P values of less than 0.05 were considered significant.

## Supplementary Material

Supplementary Figures
