# Supplementary material for: Antitumor effect of poly lactic acid nanoparticles loaded with cisplatin and chloroquine on the oral squamous cell carcinoma
Source: Aging (Albany NY). 2020 Dec 11;13(2):2593–603. doi: 10.18632/aging.202297 (PMC7880364; doi:10.18632/aging.202297)
Supplement: Supplementary Figures [file aging-13-202297-s001.pdf]

SUPPLEMENTARY FIGURES

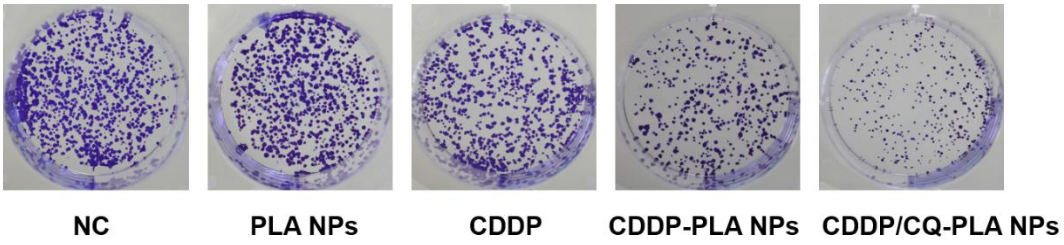

Supplementary Figure 1. CDDP/CQ-PLA NPs reduces the viability. Colony forming assays in SCC4 cells.

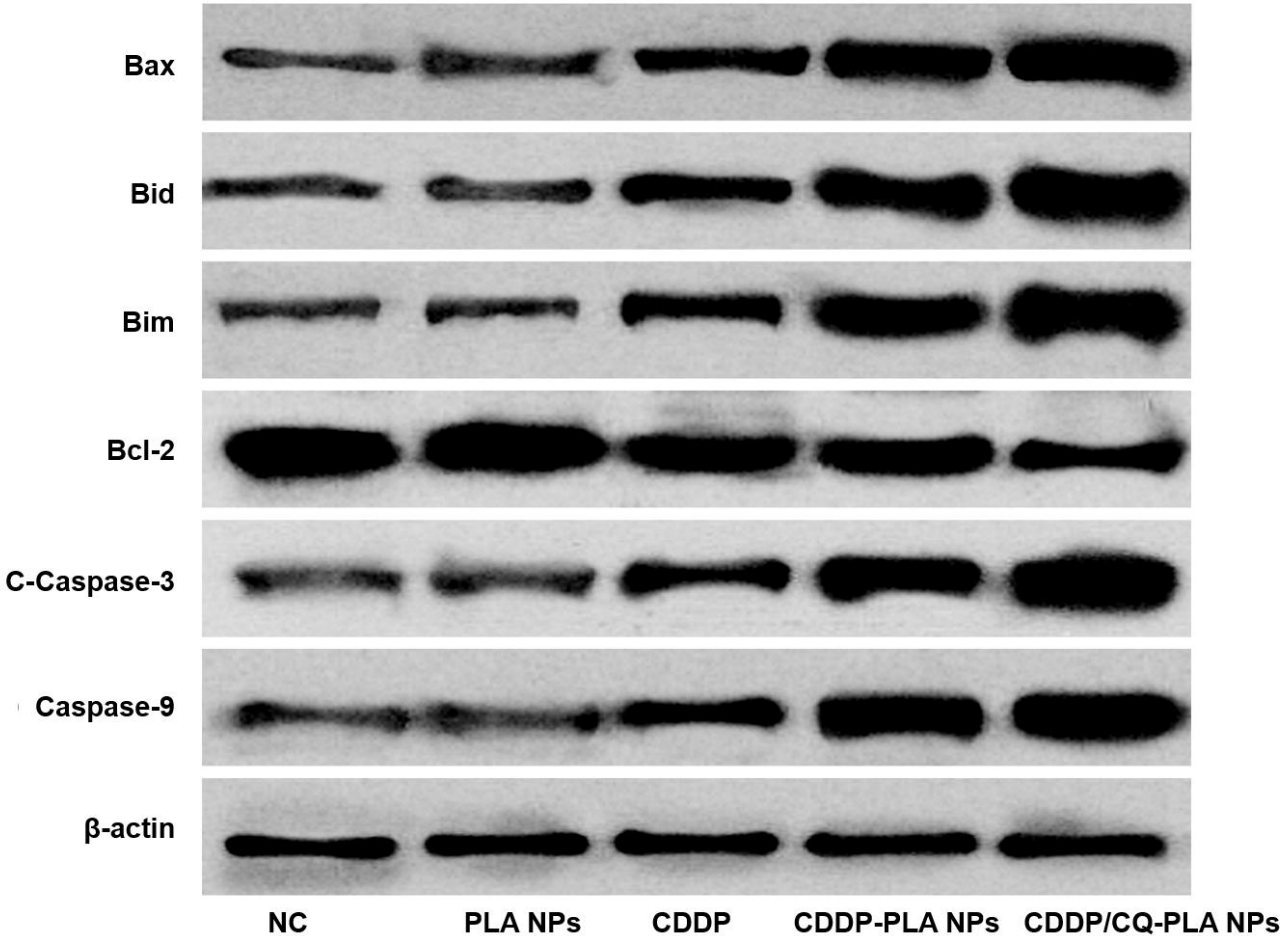

Supplementary Figure 2. CDDP/CQ-PLA NPs induces caspase-dependent apoptosis in SCC4 cells. Western blot of Bax, Bid, Bim, Bcl-2, cleaved-Caspase-3, and Caspase-9, β-actin as reference.

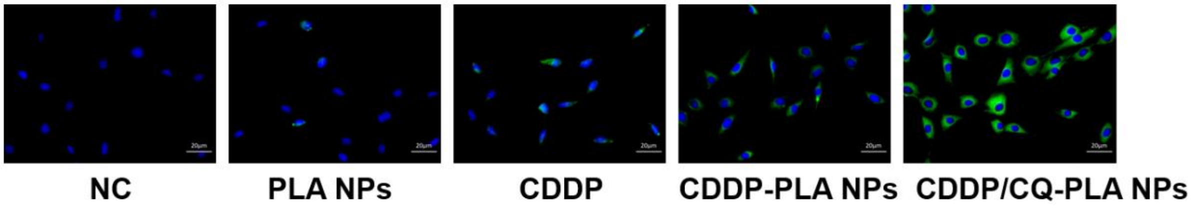

Supplementary Figure 3. CDDP/CQ-PLA NPs induces oxidative damage. ROS production in SCC4 cells.

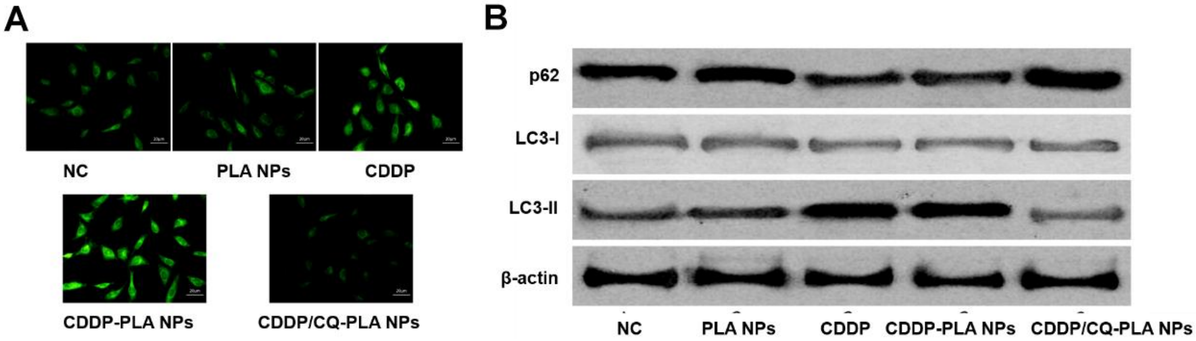

Supplementary Figure 4. CDDP/CQ-PLA NPs reduces autophagy in SCC4 cells. (**A**) Immunofluorescence confocal image of LC3; (**B**) Western blot of p62, LC3-I, and LC3-II, actin as reference.
